# Cardiovascular Risk Factors and Physical Activity for the Prevention of Cardiovascular Diseases in the Elderly

**DOI:** 10.3390/ijerph19010207

**Published:** 2021-12-25

**Authors:** Lorena Ciumărnean, Mircea Vasile Milaciu, Vasile Negrean, Olga Hilda Orășan, Stefan Cristian Vesa, Octavia Sălăgean, Silvina Iluţ, Sonia Irina Vlaicu

**Affiliations:** 1 Department 5 Internal Medicine, 4th Medical Clinic, Faculty of Medicine, “Iuliu Haţieganu” University of Medicine and Pharmacy, 400015 Cluj-Napoca, Romania; lorena_ciumarnean@yahoo.com (L.C.); mircea_milaciu@yahoo.com (M.V.M.); Vasile.Negrean@umfcluj.ro (V.N.); Hilda.Orasan@umfcluj.ro (O.H.O.); 2 Department 2 Functional Sciences, Discipline of Pharmacology, Toxicology and Clinical Pharmacology, Faculty of Medicine, “Iuliu Haţieganu” University of Medicine and Pharmacy, 400337 Cluj-Napoca, Romania; 3 Regional Institute of Gastroenterology and Hepatology ‘Octavian Fodor’ Cluj-Napoca, 400162 Cluj-Napoca, Romania; runcan.octavia@yahoo.ro; 4 Department 10 Neurosciences, Discipline of Neurology, Faculty of Medicine, “Iuliu Haţieganu” University of Medicine and Pharmacy, 400012 Cluj-Napoca, Romania; Silvina.Ilut@umfcluj.ro; 5 Department 5 Internal Medicine, 1st Medical Clinic, Faculty of Medicine, “Iuliu Haţieganu” University of Medicine and Pharmacy, 400012 Cluj-Napoca, Romania; vlaicus@yahoo.com

**Keywords:** cardiovascular disease, risk factors, elderly, prevention, physical activity

## Abstract

Cardiovascular diseases create an important burden on the public health systems, especially in the elderly, mostly because this group of patients frequently suffer from multiple comorbidities. Accumulating cardiovascular risk factors during their lifetime has a detrimental effect on an older adult‘s health status. The modifiable and non-modifiable cardiovascular risk factors are very diverse, and are frequently in a close relationship with the metabolic comorbidities of the elderly, mainly obesity and Diabetes Mellitus. In this review, we aim to present the most important cardiovascular risk factors which link aging and cardiovascular diseases, starting from the pathophysiological links between these factors and the aging process. Next, we will further review the main interconnections between obesity and Diabetes Mellitus and cardiovascular diseases of the elderly. Lastly, we consider the most important aspects related to prevention through lifestyle changes and physical activity on the occurrence of cardiovascular diseases in the elderly.

## 1. Introduction

Cardiovascular disease currently ranks first in terms of global mortality and morbidity [1]. Elderly people are more prone to developing cardiovascular diseases because age plays a key role in impairing the optimal functionality of the cardiovascular system, thus the prevalence of these diseases increases with age [2]. Conceptually, the term “elderly” usually describes a person aged 65 years or more [3], though there are studies that describe as “young elderly” the population between 60–69 years-old and as “old elderly” the persons aged 70–80 years-old [4]. In the 2019 update of the American Heart Association on Heart Disease and Stroke Statistics, the incidence of cardiovascular diseases among patients aged 40 to 60 years was on average 35–40%, in patients aged 60 to 80 years it was on average 75–78%, while in patients over 80 years of age the incidence exceeded 85% [5]. Literature is also reporting a significant difference between genders regarding the incidence of cardiovascular diseases, most likely due to the influence of sex hormones and an increase of the metabolic syndrome prevalence in women [6,7].

Regarding the risk factors for the occurrence of cardiovascular diseases, the most frequent are hypertension (double-edged sword: an intrinsically cardiovascular illness and a risk factor for other cardiovascular diseases), Diabetes Mellitus, dyslipidemia, obesity, smoking and age—these are all factors that are also involved in the development and progression of atherosclerosis [8,9].

Age is an unchangeable risk factor, also considered an independent risk factor for atherogenesis and for further cardiovascular disease [10]. However, despite the fact that there are numerous studies regarding the relationship between age and the onset of atherosclerosis, the exact mechanism directly involved in the occurrence of this condition is not known [11,12].

It is assumed that the occurrence of hypertension in older adults could be related to the fact that as physiological aging occurs, a number of changes take place at the vascular level, leading to structural and functional alterations of the vascular walls (such as increased arterial stiffness and decreased compliance) [13]. This theory is controversial, since the afore-mentioned changes are also present in young patients diagnosed with hypertension [14]. For these reasons, the acknowledgement and investigation of other risk factors associated with the aging process are required/are essential [15].

According to the US Center for Disease Control and the American Association for Prevention and Health, presence of comorbidities in which there is a state of chronic inflammation (especially Type 2 Diabetes Mellitus and obesity) and external predisposing factors (stress, sedentary lifestyle, diet and smoking) in the elderly put them at risk not only of hypertension progression, but also of the occurrence of other cardiovascular conditions [5,16]. The aforementioned risk factors can be influenced and act differently depending on the distinct genetic profile of the individuals and their age; thus, in some individuals, the risk of developing cardiovascular diseases is much higher [17]. The development of acute cardiovascular diseases in the elderly, such as acute myocardial infarction and stroke (most frequently triggered in hypertensive patients with ischemic heart disease), is a complex process in which a plethora of risk factors are involved [18,19].

In the elderly, prevention of cardiovascular disease by physical exercise plays not only a beneficial effect on physical functioning, but also in the amelioration of general quality of life [20].

This review aims to present the main pathophysiological pathways and cardiovascular risk factors linking aging and cardiovascular diseases, to explain the importance of comorbidities and to review physical exercise as a method of prevention needed to diminish the burden of cardiovascular diseases of the elderly on public health systems.

## 2. Pathophysiological Processes Encountered in Cardiovascular Diseases of the Elderly

Advanced age implies numerous functional cardiac changes, such as systolic and diastolic dysfunctions but also dysfunctions related to the electrical activity of the heart, presenting with different types of arrhythmias [21]. Over time, these changes in functionality and electrical cardiac abnormalities lead to an increase in the incidence of cardiovascular diseases in the elderly population [22].

The high number of elderly people with cardiovascular pathologies is influenced by the multiple changes that together occur with the aging process. Among the most frequent of these are a high production of oxygen free radicals (OFR), increased oxidative stress, chronic inflammation, apoptotic processes, myocardial degeneration and insulin resistance in diabetic and obese subjects [23]. The level of reactive oxygen species (ROS) builds up with age, and this increase fuels the persistence of a status of chronic systemic inflammation [23].

Elderly patients with an increased percentage of adipose tissue have considerably more chronic systemic inflammation than elderly subjects with normal body mass indexes (BMIs). The explanation for this might be the ability of the adipose tissue to secrete a number of cytokines (TNF-α, IL-6), such as resistin, which are capable of initiating and maintaining a state of chronic inflammation and insulin resistance [24,25]. Moreover, in obese patients with metabolic syndrome, a low level of adiponectin and leptin resistance was observed. Adiponectin, a peptide synthesized by adipocytes, decreases platelet aggregation and has anti-inflammatory and insulin sensitizing properties; leptin is a hormone that is involved in the inhibition of hunger, thus regulating the body’s energy balance. These changes lead to increased insulin resistance with an impact on adipose, liver and muscle tissue, and in this situation, insulin has no antilipolytic effect, leading to an increase in the production of free fatty acids and their secretion into the bloodstream. In turn, high concentrations of free fatty acids continue to fuel the state of chronic inflammation [26,27].

Enhanced production of proinflammatory factors like cytokines will lead in time to a cardiac remodeling due mainly to the extracellular matrix (ECM) reorganization at the cardiomyocite cellular level. Cardiac remodeling occurs through the cardiac accumulation of collagen fibers, secondary to changes in the expression of the tissue inhibitor of metalloproteinase and matrix metalloproteinase. Metalloproteinases are proteolytic enzymes which, when activated, destroy the ECM; thus an augmented level of metalloproteinase expression will cause a severe degradation of the matrix. The build-up of collagen fibers in the heart engenders hypertrophy of the heart muscle and eventually cardiac fibrosis [23,28]. A study by Bursenti et al. demonstrated that fibrosis and cardiac hypertrophy occurring secondary to ECM reorganization encountered in elderly patients occurs mainly in the atria, and these events are responsible for the occurrence of atrial fibrillation [29].

Normal cardiac metabolism is dependent on an adequate production of mitochondrial ATP; in the elderly, the excessive production of OFR, secondary to increased oxidative stress, impedes the normal function of mitochondria, which will resolute in cardiac dysfunction [21,30]. A study conducted in 2016 by Nakou et al. highlighted that mitochondrial DNA is highly susceptible to oxidative degradation, due to the absence of protective histones. The authors emphasized that the presence of high levels of OFR disrupts the process of mitochondrial respiration, which provokes an even greater increase in OFR levels. This vicious circle explains the exaggerated levels of oxidative stress in the myocardium of the elderly [31].

Another mechanism by which oxidative stress exerts a negative effect on the normal functionality of the heart muscle cell is by disrupting the ryanodine type 2 receptor, the main receptor involved in the release of calcium from the sarcoplasmic reticulum [32]. Since calcium ions play an essential role in the process of muscle contraction, once the ryanodine receptor is affected, it will limit the outflow of calcium ions from the sarcoplasmic reticulum, resulting in inefficient myocardial contraction and heart failure [33]. These changes were found in the vast majority of elderly people diagnosed with heart failure.

Mitochondrial damage due to an increased production of OFR is involved in the development of atherosclerosis by accelerated oxidation of plasma lipids. This was demonstrated in 2018 by an experimental study on a group of elderly mice, which were fed a diet rich in unsaturated fats (omega 6) [34]. The authors concluded that omega 6 fatty influx in aging is driving metabolic dysregulations and low-grade chronic inflammation, reinforcing the same hypothesis reviewed in 2014 by Franceschi and Campisi [35].

In addition to the events encountered in the physiological phenomenon of aging that can lead to cardiovascular diseases (increased oxidative stress, persistence of a chronic inflammation), there are many pathological conditions that lead to the precipitation of cardiovascular events, their occurrence not being strictly related to the consequences of aging or the frailty which an older adult presents. As previously mentioned, the most common pathological conditions that risk the occurrence of cardiovascular disease are Diabetes Mellitus and obesity [36].

There are many common pathophysiological pathways both for the risk factors for cardiovascular disease and for the actual cardiovascular conditions (such as atherosclerosis for Diabetes Mellitus and ischemic heart disease and the pro-inflammatory status for both obesity and myocardial infarction). These associations have many other triggers or molecular consequences which are both intricate and difficult to properly quantify when designing clinical studies on the elderly [23,25,27,36].

**Figure 1:**
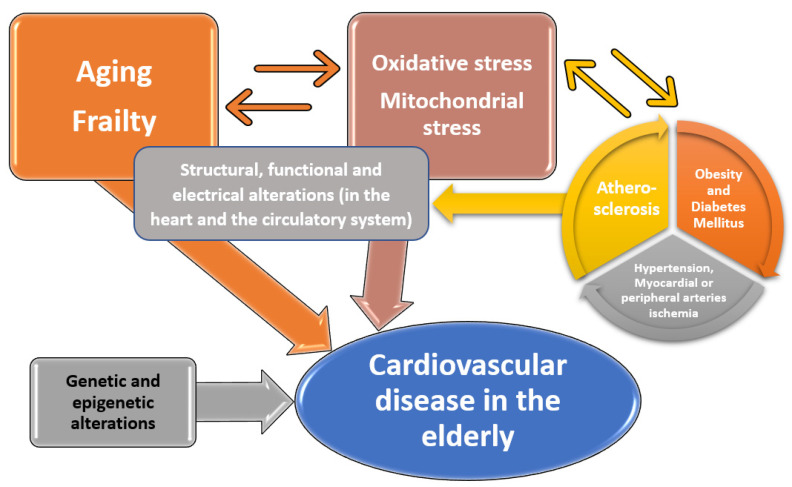
Interactions between the factors that promote the occurrence of cardiovascular diseases in the elderly.

A synthesized scheme of the interactions between the key actors that might explain the association between aging and cardiovascular disease is presented below (Figure 1, adapted after Rodgers et al. and Paneni et al.) [6,37]. In addition to the high levels of oxidative stress in the aged adult, there exists frequently the continuum Atherosclerosis obesity and Diabetes Mellitus Ischemia (which is also fueled by the oxidative stress) to promote the structural, functional and electrical alterations in the heart and the circulatory system. Another important factor, the genetic and epigenetic alterations (such as genomic instability and chromatin remodeling) were discussed in a state-of-the-art review published in 2017 by Paneni et al. [37].

## 3. Diabetes Mellitus and Cardiovascular Disease in Elderly Patients

Diabetes Mellitus is a chronic, silent, multifactorial pathology determined by the sum of several risk factors such as genetic, environmental and dietary factors. In 2020, the World Health Organization (WHO) reported an impressive increase in the incidence of diabetes: the number of patients diagnosed with this disease is 4 times higher (422 million adults) than in 1980. It is also estimated that the number of cases diagnosed by 2045 will exceed 692 million [38,39].

Type 2 Diabetes Mellitus (T2DM) is the most common form, with approximately 90% of patients diagnosed with diabetes having type 2, and over 95% of them being over 60 years old. The substrate of this pathology is an increased insulin resistance, the end result being a state of chronic hyperglycemia [40]. In the initial phase of the disease, a compensatory increase in insulin secretion occurs, and this mechanism maintains glucose homeostasis. However, as the disease progresses, pancreatic β cells undergo specific alterations, so that insulin secretion will be unable to regulate blood glucose levels [41,42].

The vast majority of patients diagnosed with T2DM have obesity, or their percentage of body fat is higher, being predominantly distributed in the abdomen; however, at the same time, obesity is considered an important risk factor in the occurrence of T2DM [43]. Mokdad et al. reported that for each additional year over the age of 60, in relation to weight, height and gender, the risk of developing T2DM increases by about 8–10% [44].

The presence of hyperglycemia, chronic inflammation and metabolic changes in diabetes puts the body under increased oxidative stress [45].

Chronic hyperglycemia, associated with other metabolic changes encountered in diabetes, predisposes the body to a series of injuries to various organs and systems, producing life-threatening complications for patients [46]. Among the most significant complications are diabetic microvascular complications (retinopathy, nephropathy) and macrovascular complications, which increase the risk of cardiovascular disease from 2 to 4 times [47,48].

Cardiovascular diseases in patients diagnosed with diabetes can materialize either in the form of hypertension, ischemic heart disease, heart failure or diabetic cardiomyopathy (DC) [49]. The latter form of manifestation is often underdiagnosed due to a long period of clinical latency, with minimal or nonspecific symptoms; although it is not easily diagnosed, the use of imaging methods, such as transthoracic or transesophageal echocardiography, improve diagnostic rates for DC [50]. DC includes the injury of cardiac muscle cells secondary to the metabolic imbalance that is inherent in diabetes, but it should be noted that these injuries occur in the absence of hypertension, valvulopathy, coronary heart disease or ischemic heart disease [51,52].

Necropsy studies performed in patients diagnosed with diabetes who died suddenly, showed structural myocardial changes representative of DC, i.e., a decrease in the number of cardiac muscle cells due to the processes of necrosis and apoptosis, changes in parasympathetic/sympathetic innervation, hypertrophy of the remaining cardiomyocytes and changes in microcirculation, but also the presence of excess amount of interstitial tissue [53,54,55].

From an echocardiographic point of view, the presentation of diabetic cardiomyopathy can be in the form of severe diastolic and systolic dysfunction (taking the form of dilated cardiomyopathy) or isolated diastolic or systolic dysfunction, but it can also emerge as an isolated left ventricular hypertrophy [56].

Other cardiovascular complications in diabetic elderly patients, such as strokes, unstable angina pectoris and acute myocardial infarction—which are complications secondary to diabetic microangiopathy—are more common among patients with long-term diabetes or patients with unbalanced T2DM. Due to the presence of diabetic neuropathy in these elderly patients, another manifestation of cardiovascular disease may be in the form of silent ischemic events [57].

Although the occurrence of T2DM in the elderly has a slightly higher incidence among males, some studies have shown that in the case of elderly women who have been diagnosed with T2DM, the risk of developing cardiovascular disease such as heart failure is higher compared to men, with an increasing mortality due to coronary heart disease [57]. Studies aiming to elucidate the causes of these differences have demonstrated that the main negative factor among women is lower serum concentrations of high-density lipoprotein (HDLc) compared to diabetic elderly men or non-diabetic women [58,59].

Regarding the mortality from cardiovascular diseases in diabetic patients, there is an increased level of evidence which states that an increased serum level of inflammatory markers is associated with a significant increase in mortality [60,61]. Thus, patients with T2DM, who have high serum levels of C-reactive protein (PCR), have an approximately 74–77% higher risk of mortality secondary to cardiovascular disease or complications of these pathologies [62].

Recent work has revealed that the risk of cardiovascular disease in people over 63–65 years, could be attributed, among other things, to the presence of a protein, namely fetuin A. Fetuin A is a glycoprotein belonging to the class of fetuins, synthesized in the liver and in adipocytes performing carrier protein functions [63]. In serum, fetuin A forms soluble, unstable complexes with calcium ions and phosphate ions. By forming these complexes, it seems that fetuin A could be involved in initiating calcium deposits (calcification) found in coronary heart disease, while having an opposite effect in peripheral arterial pathologies, namely, protection against atherosclerosis [64]. The higher risk of developing cardiovascular pathologies in the elderly with high serum levels of Fetuin-A is in fact associated with a much higher risk of developing obesity, T2DM and dyslipidemias (because fetuin A has the ability to reduce lipogenesis and to accentuate the lipolysis process) [65]. In the presence of higher concentrations of fetuin A, a diagnosis of T2DM conferred an increment of 15–20% in the incidence of cardiovascular disease, when compared to the elderly without T2DM but with elevated levels of fetuin A [63,66].

## 4. Obesity and Cardiovascular Disease in Elderly Patients

As mentioned before, age plays a role as an individual risk factor for cardiovascular diseases, because, with an advancement in age, the appearance of conditions such as obesity and T2DM becomes more common. As in the case of diabetic patients, patients with obesity can associate a status of chronic inflammation and increased oxidative stress [67].

The cardiovascular risks to an elderly patient with an increased BMI are largely influenced by blood glucose levels, plasma cholesterol levels and the presence or absence of hypertension [68]. However, in an attempt to identify the direct mechanisms involved in these combinations, it was found that in elderly patients whose blood pressure levels are maintained within normal limits, and who follow a diet in which glycemic fluctuations are not high and cholesterol levels are maintained in the reference range, the risk of developing coronary heart disease was reduced to only half of these, while three-quarters of the patients studied had a lower risk of developing stroke [69].

In light of these data, BMI should be considered an individual risk factor in the occurrence of cardiovascular disease in elderly patients and beyond [70]. To support this thesis, Fan et al. have indicated that abdominal obesity, increased perivisceral adiposity and the ratio of abdominal circumference to height are useful indicators of the risk factors for the occurrence of cardiovascular pathologies; even more, they could be considered important tools in estimating the risk of mortality secondary to cardiovascular disease [71].

Other studies have shown that the association between the advanced age of patients and the presence of excessive abdominal obesity predisposes patients to a much greater risk of developing atherosclerotic cardiovascular disease, through mechanisms incompletely elucidated so far. The same team reported that an equivalent or superunit ratio between the waist of elderly patients and their height increases the risk of cardiovascular disease by approximately 5% [72,73].

Regarding the gender difference, a slightly higher incidence of cardiovascular disease was observed among older women. This difference has been attributed to the presence of menopause among females, due to hormonal changes, especially due to decreased levels of estrogen hormones, which will increase the risk of metabolic syndrome and increase the number of complications among older, obese women at menopause [74,75].

## 5. Specific Interventions for Particular Clinical Settings

### 5.1. Stroke Prevention

Among the most daunting cardiovascular diseases, with potential life-threatening complications in the elderly, is the stroke. Stroke is one of the main causes of hospitalization, disability and death among elderly patients; cerebrovascular pathologies represent at the same time a risk factor in the development of vascular dementia [76,77].

When it comes to the prevention of strokes among older adults, several issues need to be addressed [78]: firstly, maintaining blood pressure values as close as possible to normal values, and secondly, lifestyle changes, antiplatelet therapy, regular dispensing to diagnose and monitoring of carotid artery stenosis [5,79].

Blood pressure values are especially important in elderly patients at risk of developing a stroke, because it is known that high blood pressure is the most important risk factor in the occurrence of a stroke [80]. Although prior recommendations were that in older adults, a higher blood pressure threshold was permitted (thus ensuring an efficient cerebral arterial circulation), presently studies by Framingham have exposed that the risk of stroke increases in direct proportion to the increase in blood pressure values, with a much higher incidence among elderly patients [81]. Moreover, in the case of elderly patients who died due to a stroke, the presence of hypertension was found in more than 50% of these [80,81].

Although it is not yet known what the optimal blood pressure values would be to prevent strokes in patients over the age of 70, at present there is consistent evidence that a decrease in blood pressure in elderly hypertensive patients also reduces the risk of a stroke [82].

In terms of lifestyle, one of the most harmful behaviors is smoking. Smoking is a very important risk factor when it comes to strokes. It is estimated that a smoker has an approximately 3–5 times higher risk of developing a stroke compared to a non-smoking elderly adult [83]. The World Health Federation points out that in the case of patients who have suffered a transient or ischemic stroke, over 50% are related to smoking [84].

In the case of elderly patients who have smoked, who suffered a stroke, and then gave up smoking, a decrease in the risk of a new cerebrovascular event was observed in approximately 15% of cases [85].

It is well known that a balanced diet is extremely important for all age groups. The American Heart Association (AHA) supports and recommends the adoption of a Mediterranean diet, high in fruits and vegetables, grains, fiber and white meat (fish), to reduce the risk of stroke [86]. Physical activity is just as important as diet. In patients with an average age of 70 years, physical activity of 30 min a day (even walking) reduces the risk of cardiovascular events by about 15–20% compared to sedentary patients [87].

### 5.2. Prevention of Coronary Artery Disease

Coronary heart disease includes several conditions, such as acute myocardial infarction, coronary artery failure and unstable angina pectoris. These conditions are among the leading causes of mortality and morbidity among elderly patients. However, age predisposes to coronary heart disease regardless of the presence or absence of other risk factors. Nonetheless, in the presence of additional risk factors, such as family history of coronary heart disease, menopause, dyslipidemia, sedentary lifestyle, tobacco use, the presence of obesity or T2DM, an incidence of coronary heart disease of up to 25% higher was observed [88].

Regarding the prevention of coronary artery disease, it is known that in the case of hypertensive patients, especially in the elderly, effective antihypertensive medication diminishes the risk of coronary heart disease or a stroke [89].

Smoking or tobacco use in any form is strongly associated with the appearance of many pathologies, such as malignancies and cardiovascular diseases, especially coronary heart disease and acute myocardial infarction. A study performed on a group of patients who were over 65 years of age, had coronary artery disease and smoked showed that the mortality of these patients was 23–30% higher compared to the control group consisting of elderly patients with coronary heart disease but who did not smoke [90]. In the case of elderly patients who smoke and who have suffered an acute myocardial infarction, smoking cessation reduces the risk of the recurrence of cardiac events, so that approximately 3 years after quitting smoking, the risk of potentially fatal vascular events is equal to that of non-smoking patients [91].

Regarding dietary measures, the debate is still ongoing about the benefits of certain dietary interventions in the prevention of coronary heart disease. The most studied measure, the Mediterranean diet, proved a useful protector against ischemic heart disease occurrence. However, randomized studies have established that replacing saturated fats with vegetable oils rich in linoleic acid lowered serum cholesterol levels, but without a concurrent reduction regarding the risk of death from ischemic coronary heart disease [92].

Physical activity reduces the risk of coronary heart disease in older adults. A study conducted in the Netherlands on patients aged 65–70 years concluded that even housework or cycling reduces the risk of coronary events and death from coronary heart disease [93].

Regarding lipid-lowering agents, a body of evidence suggested that statins administered for primary prevention in patients aged 65 to 74 years (with hypertension and moderate dyslipidemia) do not confer any benefits, and in patients over 75 years, the benefits are insignificant [94]. However, a 2021 systematic review conducted by Awad et al. concluded that statin use in older adults aged ≥65 years was associated by a 14% lower risk for mortality (all-cause) and a 20% reduction of the risk of cardiovascular death [95].

### 5.3. Prevention of Peripheral Artery Disease

Peripheral arterial disease is characterized by gradual and chronic narrowing of the arterial lumen, which results in inadequate and inefficient irrigation of tissues. Clinical manifestations are characterized by acute or chronic ischemia, translated as pain in the territory served by the artery, with reduced lumen, functional impotence and, finally, the occurrence of tissue necrosis [96].

The frequency of peripheral arterial diseases increases with age, so that at the age of 80–90 years, the incidence of this pathology peaks, being about 15–20% higher than in other age groups. Due to the most often silent and prolonged development of peripheral vascular disease, patients go to the doctor in the final stages of the disease, the only solution for them usually being amputation. Among the risk factors in its occurrence in addition to age are tobacco use, dyslipidemia, neglected hypertension and diabetes [97].

As is the case for the aforementioned pathologies, a good control of hypertension, diabetes, smoking cessation and a diet rich in fiber of plant origin are the keys to success in terms of peripheral arterial disease [98].

In patients who have developed this condition, lifestyle changes, smoking cessation, exercise (or even walking for 30 min each day) and treatment with statins can lead to a slowdown in the pathophysiological process and a slight improvement in the quality of life of these patients [99]. Additionally, for this category of patients, regular consultations with the cardiologist and periodic Doppler ultrasounds are indicated in order to establish an early diagnosis and correctly determine the evolutionary stage of this disease [100]. If patients are symptomatic, have intermittent claudication or other manifestations of peripheral arterial disease, antiplatelet therapy is recommended to minimize the risk of debilitating or potentially fatal cardiovascular events such as acute myocardial infarction, sudden death or strokes. Antiplatelet therapy with Aspirin or Clopidogrel is preferred. No benefit was observed in the concomitant administration of the two preparations, but an increase in the risk of bleeding was observed, which is why the combination is not recommended [101].

In elderly people with peripheral artery disease, which is often associated with hypertension and ischemic heart disease, a key factor which ameliorates the quality of life is good treatment adherence. Non-adherence to treatment in the elderly can lead to further aggravation of their physical or mental symptoms, together with a further burden on health systems and caregivers [102].

## 6. Physical Activity in the Elderly and the Prevention of Cardiovascular Diseases

Cardiovascular disease prevention in the general population, and especially in older adults, starts from lifestyle modification [76]. Because studies on dietary interventions, stopping smoking and alcohol consumption particularly have already accumulated a large body of evidences at present, these will make the object of a further review.

To prevent cardiovascular disease not just in elderly patients, but in all age groups, it is recommended to avoid a sedentary lifestyle and to exercise within the limits of individual tolerance [87]. There is a lack of precision-designed studies to evaluate properly the differences between certain interventions in particular clinical settings and in different age sub-groups. The literature needs better standardized studies to evaluate which is the best strategy to diminish cardiovascular risk using preventive measures (such as physical activity regimen or dietary patterns). These preventive measures need to be studied in every particular clinical setting, in order to finally be able to prescribe a tailored intervention for a certain type of patient. 

It is common knowledge that physical exercise has a positive impact on improving health and quality of life. In the elderly, regular exercise can exert an important contribution in reducing falls, maintaining good physical function and maintaining a good quality of life [103].

### 6.1. Mechanisms and Effects

There are multiple studies that aimed to establish the mechanisms by which physical activity has a positive impact on health status of the elderly, the benefits being already reviewed since the early 2000s [104]. Besides improving of the physiologic cardiovascular parameters, physical exercise improves cardiovascular symptoms (especially related to heart failure) and blood pressure, and it decreases the risk of coronary artery disease, together with improving the lipid profile and insulin sensitivity [103,104,105]. In addition, physical exercise helps to reduce sympathetic activity, achieving better blood pressure and heart-rate control [105,106,107].

The cardiovascular benefits gained by the elderly due to regular physical exercise are also linked with an increased general quality of life, because it is proven at present that wellbeing and cognitive functioning are also improved in the elderly who exercise regularly [108].

**Figure 2:**
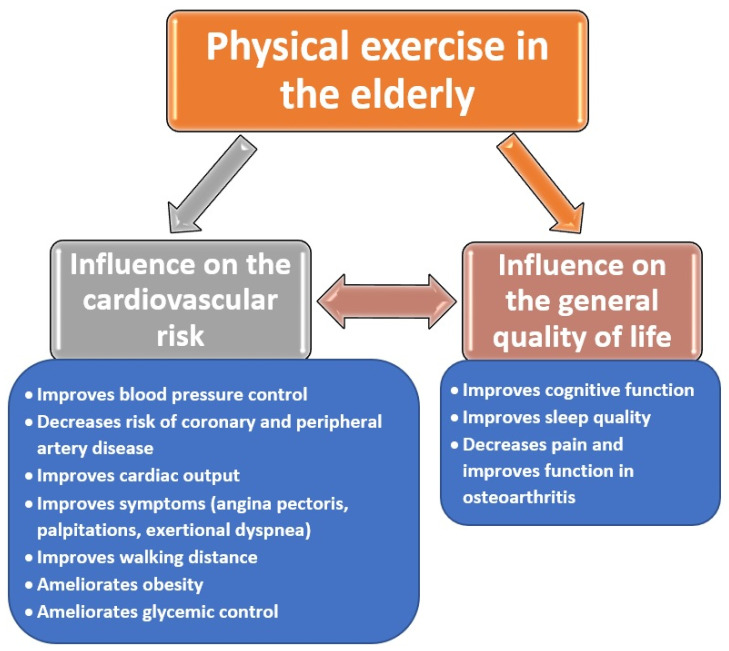
Influence of physical exercise on the cardiovascular risk and quality of life of the elderly [104,105,106,107,108].

The effects of physical exercise on cardiovascular risk factors and the quality of life of the elderly are highlighted in the following figure (Figure 2).

### 6.2. Types of Physical Activities Studied in the Elderly

The key principles of the recommended physical activity in older adults are the following: the preferred exercises are aerobic ones, which need to be supplemented by balance, strengthening and flexibility exercises [109]. Both the British National Health Service NHS and the American Center for Disease Control and Prevention CDC recommend that adults above the age of 65 should perform at least 150 min of moderate intensity physical activity each week (the best example being brisk walking at 30 min per day, 5 days a week) [110,111]. If the elderly are already active persons, the physical activity can be limited to 75 min of vigorous intensity activity per week (such as jogging and running). At least 2 days a week, the subjects must add to the previous examples some muscle-strengthening activities that work all major muscle groups (hips, legs, back, abdomen, shoulders, chest and arms) [110,111]. In a recently published nationwide cohort study on older adults, physical activity was inversely associated with long-term mortality risk, confirming that the maximum benefits in terms of survival were achieved at around 150 min of exercise per week [112].

Many studies emphasize that there should not exist an ideal exercise to be prescribed in a certain profile of the older adult, because it was shown that even modest levels of physical activity offer benefits in terms of cardiovascular risk, compared to being completely inactive [113]. To date, the most commonly recommended exercise is walking, and gait speed is proposed as a powerful marker of longevity in elderly and frail subjects [114]. Walking counts as a moderate-intensity activity, as are water aerobics, hiking (flat terrain), dancing, riding a bike or pushing a lawn mower [110]. Vigorous activities can include any of the following: running or swimming (non-professional level), aerobics, tennis, football or hiking uphill [110]. For muscle strengthening, it is recommended to work with resistance bands (such as Thera-Band^®^ elastic bands), lifting weights (even carrying shopping bags) or yoga and Pilates exercises [110,115]. Older women are more predisposed to practice less physical exercise than men. In a recently published study, it was shown that general exercises and dancing improve balancing skills, and might reduce the risk of falls in elderly women [116].

Unfortunately, during the COVID-19 pandemic, older adults have suffered a further reduction of physical exercise, due to social distancing. At present, there is low to moderate evidence that mobile or internet-delivered (eHealth) interventions may be efficient in promoting and increasing the levels of physical activity in the elderly [117].

### 6.3. Physical Activity, Insulin Sensitivity and Glycemic Control

Regardless of the exercise performed, whether it is running or walking, many studies have reported that during the active contraction of striated muscles, there is an increase in the translocation of type 4 glucose transporter (GLUT4) from the cytoplasm of striated muscle cells, the result being the promotion of blood glucose absorption, thus improving insulin resistance; this mechanism is known to play a major role in the development of T2DM [118,119]. After glucose enters the cells, it will be transformed into glucose-6-phosphate (G-6-P) through the process of phosphorylation under the action of hexokinase. G-6-P is directly involved in the processes of glycolysis and glycogenesis, processes that increase the level of blood glucose absorption. A study performed on diabetic rats illustrated how physical activity leads to an increase in G-6-P levels in striated muscle cells but also to improved activity of GLUT4, hexokinase and glycogen-synthetase activity, thereby improving glucose tolerance and lowering blood glucose levels. Reduction of blood glucose levels is synonymous with an optimal control of diabetes, hence the risk of cardiovascular disease secondary to poorly controlled diabetes an unbalanced diabetes among elderly patients will be significantly lowered [120].

### 6.4. Physical Activity and Blood Pressure Values

As previously mentioned, high blood pressure predisposes a person to cardiovascular disease, as well as complications such as acute myocardial infarction, heart failure or stroke [121]. Following physical activity, a transient decrease in blood pressure was found, both among people diagnosed with hypertension and among normotensive people [122].

Studies which determined the beneficial effect of physical activity on blood pressure proved that physical activity maintained over a long period of time leads to a decrease in systolic blood pressure by about 5 mmHg, and a reduction in diastolic blood pressure by about 3.5 mmHg. It is well known that a reduction in systolic blood pressure values by 2–3 mmHg decreases the risk of death secondary to a stroke by about 5%, and secondary to an acute coronary episode by 3–4%. However, the decrease in blood pressure is a transient one, which is why performing physical exercises, such as walking, must be performed daily [123,124]. 

Nonetheless, when performing exercises of much higher intensity, and without these being adjusted to age and muscle mass, opposite effects occur, namely increased vasoconstrictor activity, an increase in oxidative stress and an increase in blood pressure. Because of these observations, light physical activity is recommended at present for the elderly, such as walking for 30 min daily [125].

## 7. Conclusions

In conclusion, cardiovascular diseases remain a major cause of disability or mortality among elderly patients, age being a major risk factor in their occurrence. This role is accentuated by the increased probability that, in the case of this category of patients, and in addition to old age and frailty, there is an impressive number of other factors, such as obesity, smoking, sedentary lifestyle or pathologies such as diabetes mellitus and hypertension.

However, as mentioned above, in the case of accumulating risk factors, their detection and treatment is crucial in the subsequent evolution of these patients. By treating them, changing their lifestyle and exercising regularly, the occurrence of cardiovascular diseases can be delayed or their progression might be slowed down.

Physical activity plays an important role in maintaining a good physical functioning and improving the quality of life of the elderly. Better-designed studies are needed to evaluate the particularities of each specific clinical setting, in order to help doctors to prescribe a tailored schedule of physical activity for a certain type of patient.

## References

[1] Galiuto L., Locorotondo G. (2017). Cardiovascular Aging. Integrative Cardiology.

[2] Seco M., Edelman J.J.B., Forrest P., Nig M., Wilson M.K., Fraser J., Bannon P.G., Vallely M.P. (2014). Geriatric cardiac surgery: Chronology vs. biology. Heart Lung Circ..

[3] Singh S., Bajorek B. (2014). Defining ‘elderly’ in clinical practice guidelines for pharmacotherapy. Pharm. Pract..

[4] Milanović Z., Pantelić S., Trajković N., Sporiš G., Kostić R., James N. (2013). Age-related decrease in physical activity and functional fitness among elderly men and women. Clin. Interv. Aging.

[5] Benjamin E.J., Muntner P., Bittencourt M.S., Callaway C.W., Carson A.P., Chamberlain A.M., Chang A.R., Cheng S., Das S.R., Delling F.N. (2019). Heart Disease and Stroke Statistics-2019 Update: A Report From the American Heart Association. Circulation.

[6] Rodgers J.L., Jones J., Bolleddu S.I., Vanthenapalli S., Rodgers L.E., Shah K., Karia K., Panguluri S.K. (2019). Cardiovascular Risk Associated with Gender and Aging. J. Cardiovasc. Dev. Dis..

[7] Faulkner J.L., Belin De Cantemele E.J. (2019). Sex hormones, aging and cardiometabolic syndrome. Biol. Sex Differ..

[8] Garcia M., Mulvagh S.L., Noel Bairey Merz C., Buring J.E., Manson J.E. (2016). Cardiovascular Disease in Women: Clinical Perspectives. Circ. Res..

[9] Keto J., Ventola H., Jokelainen J., Linden K., Keinanen-Kiukaanniemi S., Timonen M., Ylisaukko-oja T., Auvinen J. (2016). Cardiovascular disease risk factors in relation to smoking behavior and history: A population- based cohort study. Open Heart.

[10] North B.J., Sinclair V.A. (2012). The intersection between aging and cardiovascular disease. Circ. Res..

[11] Yazdanyar A., Newman A.B. (2009). The burden of cardiovascular disease in the elderly: Morbidity, mortality and costs. Clin. Geriatr. Med..

[12] Spannella F., Di Pentima C., Giulietti F., Buscarini S., Giordano P., Sarzani R. (2020). Prevalence of Sublinical Carotid Atherosclerosis and Role of Cardiovascular Risk Factors in Older Adults: Atherosclerosis and Aging are Not Synonyms. High Blood Press Cardiovasc Prev..

[13] Bruno R.M., Masi S., Taddei M., Taddei S., Virdis A. (2018). Essential Hypertension and Dunctional Microvascular Ageing. High Blood Press. Cardiovasc. Prev..

[14] Nilsson P.M. (2008). Early vascular aging (EVA): Consequences and prevention. Vasc. Health Risk Manag..

[15] Hay M., Barnes C., Huentelman M., Brinton R., Ryan L. (2020). Hypertension and Age-Related Cognitive Impairment: Common Risk Factors and a Role for Precision Aging. Curr. Hypertens. Rep..

[16] Dhingra R., Vasan R.S. (2012). Age as a cardiovascular risk factor. Med. Clin. N. Am..

[17] Iorga A., Cunningham C.M., Moazeni S., Ruffenach G., Umar S., Eghbali M. (2017). The protective role of estrogen and estrogen receptors in cardiovascular disease and the controversal use of estrogen therapy. Biol. Sex Differ..

[18] Wang Y., Han H.R., Yang W., Zhang H., Zhang J., Ruan H., Tang N., Ren J., Sun X., Li C. (2021). Association between risk factors for cardiovascular disease and frialty among community-dwelling older adults in Lanzhou, China. Int. J. Nurs. Sci..

[19] Yatsuya H., Matsunaga M., Li Y., Ota A. (2017). Risk Factor of Cardiovascular Disease Among Older Individuals. J. Atheroscler. Thromb..

[20] Buchner D.M. (2009). Physical activity and prevention of cardiovascular disease in older adults. Clin. Geriatr. Med..

[21] Steeman M., Lande G. (2017). Cardiac aging and heart disease in humans. Biophys. Rev..

[22] Curtis A.B., Karki R., Hattoum A., Sharma U.C. (2018). Arrhythmias in Patients ≥80 Years of Age: Pathophysiology, Management, and Outcomes. J. Am. Coll. Cardiol..

[23] Meschiari C.A., Ero O.K., Pan H., Finkel T., Lindsey M.L. (2017). The impact of aging on cardiac extracellular matrix. Geroscience.

[24] Meneses M.J., Silvester R., Sousa-Lima I., Macedo M.P. (2019). Paraoxonase-1 as a Regulator of Glucose and Lipid Homeostasis: Impact on the Onset and Progression of Metabolic Disorders. Int. J Mol. Sci..

[25] Merino J., Udler M.S., Leong A., Meigs J.B. (2017). A Decade of Genetic and Metabolomic Contributions to Type 2 Diabetes Risk Prediction. Curr. Diab. Rep..

[26] Zhou M., Liu X.H., Liu Q.Q., Chen M., Bai H., Jiang C.Y., Guan L.B., Fan P. (2021). Lactonase activity and status of paraoxonase-1 and oxidative stress in neonates of women with gestational diabetes mellitus. Pediatr. Res..

[27] Artasensi A., Pedretti A., Vistoli G., Fumagalli L. (2020). Type 2 Diabetes Mellitus: A Review of Multi-Target Drugs. Molecules.

[28] Martos R., Baugh J., Ledwidge M.O., Loughlin C., Conlon C., Patle A., Donnelly S.C., McDonald K. (2007). Diastolic heart failure: Evidence of increased myocardial collagen turnover linked to diastolic dysfunction. Circulation.

[29] Burstein B., Nattel S. (2008). Atrial fibrosis: Mechanisms and clinical relevance in atrial fibrillation. J. Am. Coll. Cardiol..

[30] Martin-Fernandes A., Gredilla R. (2016). Mitochondria and oxidative stress in heart aging. Age (Dordr).

[31] Nakou E.S., Parthenakis F.I., Kallergis E.M., Marketou M.E., Nakos K.S., Vardas P.E. (2016). Healthy aging and myocardium: A complicated process with various effects in cardiac structure and physiology. Int. J. Cardiol..

[32] Xie W., Santulli G., Reiken S.R., Yuan Q., Osborne B.W., Chen B.X., Mark A.R. (2015). Mitochondrial oxidative stress promotes atrial fibrillation. Sci. Rep..

[33] Babušíková E., Lehotský J., Dobrota D., Račay P., Kaplán P. (2012). Age-associated changes in Ca(2+)-ATPase and oxidative damage in sarcoplasmic reticulum of rat heart. Physiol. Res..

[34] Kain V., Ingle K.A., Kachman M., Baum H., Shanmugam G., Rajasekaran N.S., Young M.E., Halade G.E. (2018). Excess ω-6 fatty acids influx in aging drives metabolic dysregulation, electrocardiographic alterations, and low-grade chronic inflammation. Am. J. Physiol. Heart Circ. Physiol..

[35] Franceschi C., Campisi J. (2014). Chronic inflammation (inflammaging) and its potential contribution to age-associated diseases. J. Gerontol. A Biol. Sci. Med. Sci..

[36] Lu Y., Hajifathalian K., Ezzati M., Woodward M., Rimm E.B., Danaei G. (2014). Metabolic mediators of the effects of body-mass index, overweight, and obesity on coronary heart disease and stroke: A pooled analysis of 97 prospective cohorts with 1.8 million participants. Lancet.

[37] Paneni F., Diaz Canestro C., Libby P., Luscher T.F., Camici G.G. (2017). The Aging Cardiovascular System: Understanding It at the Cellular and Clinical Levels. J. Am. Coll. Cardiol..

[38] Haldar S.R., Chakrabarty A., Chowdhury S., Haldar A., Sengupta S., Bhattacharyya M. (2015). Oxidative stress-related genes in type 2 diabetes: Association analysis and their clinical impact. Biochem. Genet..

[39] Chatterjee S., Khunti K., Davies M.J. (2017). Type 2 diabetes. Lancet.

[40] Galicia-Garcia U., Benito-Vicente A., Jebari S., Lerrea-Sebal A., Siddiqi H., Uribe K.B., Ostolaza H., Martín C. (2020). Pathophysiology of Type 2 Diabetes Mellitus. Int. J. Mol. Sci..

[41] Dimas A.S., Lagou V., Barker A., Knowles J.W., Magi R., Hivert M.F., Benazzo A., Rybin D., Jackson A.U., Stringham H.M. (2014). Impact of type 2 diabetes susceptibility variants on quantitative glycemic traits reveals mechanistic heterogeneity. Diabetes.

[42] Florez J.C. (2016). Leveraging Genetics to Advance Type 2 Diabetes Prevention. PLoS Med..

[43] Leitner D.R., Fruhbeck G., Yumuk V., Schindler K., Micic D., Woodward E., Toplak H. (2017). Obesity and Type 2 Diabetes: Two Diseases with a Need for Combined Treatment Strategies—EASO Can Lead the Way. Obes. Fact..

[44] Mokdad A.H., Ford E.S., Bowman B.A., Dietz W.H., Vincor F., Bales V.S., Mark J.S. (2003). Prevalence of obesity, diabetes, and obesity-related health risk factors. JAMA.

[45] Oguntibeju O.O. (2019). Type 2 diabetes mellitus, oxidative stress and inflammation: Examining the links. Int. J. Physiol. Pathophysiol. Pharmacol..

[46] Zadhoush F., Sadeghi M., Pourfarzam M. (2015). Biochemical changes in blood of type 2 diabetes with and without metabolic syndrome and their association with metabolic syndrome components. J. Res. Med. Sci..

[47] Papatheodorou K., Banach M., Bekiari E., Rizzo M., Edmonds M. (2018). Complications of Diabetes. J. Diabetes Res..

[48] Silva E.F.F., Memdes Ferreira C.M., De Pinho L. (2017). Risk factors and complications in type 2 diabetes outpatients. Rev. Assoc. Med. Bras. (1992).

[49] Halter J.B., Musi N., Horne McFarland F., Crandall J.P., Goldberg A., Harkless L., Hazzard W.R., Huang E.S., Kirkman M.S., Plutzky J. (2014). Diabetes and cardiovascular disease in older adults: Current status and future directions. Diabetes.

[50] Athithan L., Gulsin G.S., McCann G.P., Levelt E. (2019). Diabetic cardiomyopathy: Pathophysiology, theories and evidence to date. World J. Diabetes.

[51] Kaur N., Guan Y., Raja R., Ruiz-Velasco A., Liu W. (2021). Mechanisms and Therapeutic Prospects of Diabetic Cardiomyopathy Through the Inflammatory Response. Front. Physiol..

[52] Teodebusch R., Belenchia A., Pulakat L. (2018). Diabetic Cardiomyopathy: Impact of Biological Sex on Disease Development and Molecular Signatures. Front. Physiol..

[53] Jia G., Hill A.M., Sowers J.R. (2018). Diabetic cardiomyopathy: An update of mechanisms contributing to this clinical entity. Circ. Res..

[54] Miki T., Yuda S., Kouzu H., Miura T. (2013). Diabetic cardiomyopathy: Pathophysiology and clinical features. Heart Fail. Rev..

[55] Seferovic P.M., Petrie M.C., Filippatos G.S., Anker S.D., Rosano G., Bauersachs J., Paulus W.J., Komajda M., Cosentino F., A de Boer R. (2018). Type 2 diabetes mellitus and heart failure: A position statement from the Heart Failure Association of the European Society of Cardiology. Eur. J. Heart Fail..

[56] Paolillo S., Marsico F., Prastaro M., Renga F., Esposito L., De Martino F., Di Napoli P., Esposito I., Ambrosio A., Ianniruberto M. (2019). Diabetic Cardiomyopathy: Definition, Diagnosis, and Therapeutic Implications. Heart Fail. Clin..

[57] Li C.L., Chiu Y.C., Chang H.Y., Hsu K.H., Bai Y.B., Wang H.H. (2013). Association of geriatric conditions and cardiovascular diseases with disability in older adults with diabetes: Findings from a nationally representative survey. Geriatr. Gerontol. Int..

[58] Kautzky-Willer A., Harreiter J., Pacini G. (2016). Sex and Gender Differences in Risk, Pathophysiology and Complications of Type 2 Diabetes Mellitus. Endocr. Rev..

[59] Lee P.G., Halter J.B. (2017). The Pathophysiology of Hyperglycemia in Older Adults: Clinical Considerations. Diabetes Care.

[60] Liu C., Feng X., Li Q., Wang Y., Li Q., Hua M. (2016). Adiponectin, TNF-α and inflammatory cytokines and risk of type 2 diabetes: A systematic review and meta-analysis. Cytokine.

[61] El Assar M., Angulo J., Rodriguez-Manas L. (2016). Diabetes and ageing-induced vascular inflammation. J. Physiol..

[62] Tian R., Tian M., Wang L., Qian H., Zhang S., Pang H., Liu Z., Fang L., Shen Z. (2019). C-reactive protein for predicting cardiovascular and all-cause mortality in type 2 diabetic patients: A meta-analysis. Cytokine.

[63] Jensen M.K., Bartz T.M., Mukamal K.J., Djousse L., Kizer J.R., Tracy R.P., Zieman S.J., Rimm E.B., Siscovick D.S., Shlipak M. (2013). Fetuin-A, type 2 diabetes, and risk of cardiovascular disease in older adults: The cardiovascular health study. Diabetes Care.

[64] Icer M.A., Yıldıran H. (2021). Effects of fetuin-A with diverse functions and multiple mechanisms on human health. Clin. Biochem..

[65] Mancio J., Barros A.S., Conceicao G., Pessoa-Amorim G., Santa C., Bartosch C., Ferreira W., Carvalho M., Ferreira N., Vouga L. (2020). Epicardial adipose tissue volume and annexin A2/fetuin-A signalling are linked to coronary calcification in advanced coronary artery disease: Computed tomography and proteomic biomarkers from the EPICHEART study. Atherosclerosis.

[66] Eleftheriadou I., Grigoropoulou P., Kokkinos A., Mourouzis I., Perrea D., Katsilambros N., Sfikakis P.P., Tentolouri N. (2017). Association of plasma fetuin-a levels with peripheral arterial disease and lower extremity arterial calcification in subjects with type 2 diabetes mellitus. J. Diabetes Complicat..

[67] Marseglia L., Manti S., D’Angelo G., Nicotera A., Parisi E., Di Rosa G., Gitto E., Arrigo T. (2014). Oxidative stress in obesity: A critical component in human diseases. Int. J. Mol. Sci..

[68] Roh H.T., Cho S.Y., So W.Y. (2017). Obesity promotes oxidative stress and exacerbates blood-brain barrier disruption after high-intensity exercise. J. Sport Health Sci..

[69] Dudina A., Cooney M.T., De Bacquer D., De Backer G., Ducimetiere P., Jousilahti P., Keil K., Menotti A., Njølstad I., Oganov R. (2011). Relationships between body mass index, cardiovascular mortality, and risk factors: A report from the SCORE investigators. Eur. J. Cardiovasc. Prev. Rehabil..

[70] Khosravi A., Tabib A.A., Golshadi I., Siadat Z.D., Bahonar A., Zarfeshani S. (2012). The Relationship between Weight and CVD Risk Factors in a Sample Population from Central Iran (Based on IHHP). ARYA Atheroscler..

[71] Fan H., Li X., Zheng L., Chen X., Lan Q., Wu H., Ding X., Qian D., Shen Y., Yu Z. (2016). Abdominal obesity is strongly associated with Cardiovascular Disease and its Risk Factors in Elderly and very Elderly Community-dwelling Chinese. Sci. Rep..

[72] Schneider H.J., Friedrich N., Klotsche J., Pieper L., Nauck M., John U., Dörr M., Felix S., Lehnert H., Pittrow D. (2010). The predictive value of different measures of obesity for incident cardiovascular events and mortality. J. Clin. Endocrinol. Metab..

[73] Batsis J.A., Zagaria A.B. (2018). Addressing Obesity in Aging Patients. Med. Clin. N. Am..

[74] Decaria J.E., Sharp C., Petrella R.J. (2012). Scoping review report: Obesity in older adults. Int. J. Obes. (Lond)..

[75] Maas A.H.E.M., Appelman Y.E.A. (2010). Gender differences in coronary heart disease. Neth. Heart J..

[76] Coll P.P., Roche V., Olsen J.S., Voit J.H., Bowen E., Kumar M. (2020). The Prevention of Cardiovascular Disease in Older Adults. J. Am. Geriatr. Soc..

[77] Roth G.A., Johnson C., Abajobir A., Abd-Allah F., Abera S.F., Abyu G., Ahmed M., Aksut B., Alam T., Alam K. (2017). Global, Regional, and National Burden of Cardiovascular Diseases for 10 Causes, 1990 to 2015. J. Am. Coll. Cardiol..

[78] Forti P., Maioli F., Procaccianti G., Nativio V., Lega M.V., Coveri M., Zoli M., Sacquegna T. (2013). Independent predictors of ischemic stroke in the elderly: Prospective data from a stroke unit. Neurology.

[79] Howard G., Goff D.C. (2012). Population shifts and the future of stroke: Forecasts of the future burden of stroke. Ann. N. Y. Acad. Sci..

[80] Pase M.P. (2012). Modifiable vascular markers for cognitive decline and dementia: The importance of arterial aging and hemodynamic factors. J. Alzheimers Dis..

[81] Seshadri S., Wolf P.A. (2007). Lifetime risk of stroke and dementia: Current concepts, and estimates from the Framingham Study. Lancet Neurol..

[82] Garrison S.R., Kolber M.R., Korownyk C.S., McCracken R.K., Heran B.S., Allan G.M. (2017). Blood pressure targets for hypertension in older adults. Cochrane Database Syst. Rev..

[83] Cornelius M.E., Wang T.W., Jamal A., Loretan C.G., Neff L.J. (2020). Tobacco Product Use Among Adults—United States, 2019. MMWR Morb. Mortal Wkly Rep..

[84] Bennett D.A., Krishnamurthi R.V., Barker-Collo S., Forouzanfar M.H., Naghavi M., Connor M., Lawes C.M.M., Moran A.E., AndersonM L.M., Roth G.A. (2014). The global burden of ischemic stroke: Findings of the GBD 2010 study. Glob. Heart.

[85] Chen J., Li S., Zhend K., Wang H., Xie Y., Xu P., Dai Z., Gu M., Xia Y., Zhao M. (2019). Impact of Smoking Status on Stroke Recurrence. J. Am. Heart Assoc..

[86] Paterson K.E., Myint P.K., Jennings A., Bain L.K.M., Lentjes M.A.H., Khaw K.T., Welch A.A. (2018). Mediterranean Diet Reduces Risk of Incident Stroke in a Population with Varying Cardiovascular Disease Risk Profiles. Stroke.

[87] Rippe J.M. (2019). Lifestyle Strategies for Risk Factor Reduction, Prevention, and Treatment of Cardiovascular Disease. Am. J. Lifestyle Med..

[88] Arnett D.K., Bluementhal R.S., Albert M.A., Buroker A.B., Goldberger Z.D., Hahn E.J., Himmelfarb C.D., Khera A., Lloyd-Jones D., McEvoy J.W. (2019). 2019 ACC/AHA Guideline on the Primary Prevention of Cardiovascular Disease: A Report of the American College of Cardiology/American Heart Association Task Force on Clinical Practice Guidelines. Circulation.

[89] Blood Pressure Lowering Treatment Trialists Collaboration (2021). Pharmacological blood pressure lowering for primary and secondary prevention of cardiovascular disease across different levels of blood pressure: An individual participant-level data meta-analysis. Lancet.

[90] Teo K.K., Ounpuu S., Hawken S., Pandey M.R., Valentin V., Hunt D., Diaz R., Rashed W., Freeman R., Jiang L. (2006). Tobacco use and risk of myocardial infarction in 52 countries in the INTERHEART study: A case-control study. Lancet.

[91] Johanneke Van Den Berg M., Graaff Y., Deckers J.W., De Kanter W., Algra A., Kappelle L.J., de Borst G.J., Cramer M.J.M., Visseren F.L.J., SMART study group (2019). Smoking cessation and risk of recurrent cardiovascular events and mortality after a first manifestation of arterial disease. Am. Heart J..

[92] Ramsden C.E., Zamora D., Majchzak-Hong S., Faurot K.R., Broste S.K., Frantz R.P., Davis J.M., Ringel A., Suchindran C.M., Hibbeln J.R. (2016). Re-evaluation of the traditional diet-heart hypothesis: Analysis of recovered data from Minnesota Coronary Experiment (1968–1973). BMJ.

[93] Koolhaas C.M., Dhana K., Golubic R., Schoufour J.D., Hofman A., Rooij F.J.A., Franco O.H. (2016). Physical Activity Types and Coronary Heart Disease Risk in Middle-Aged and Elderly Persons: The Rotterdam Study. Am. J. Epidemiol..

[94] Han B.H., Sutin D., Williamson J.D., Davis B.R., Piller L.B., Pervin H., Pressel S.P., Blaum C.S., ALLHAT Collaborative Research Group (2017). Effect of Statin Treatment vs Usual Care on Primary Cardiovascular Prevention Among Older Adults: The ALLHAT-LLT Randomized Clinical Trial. JAMA Intern. Med..

[95] Awad K., Mohammed M., Zaki M.M., Abushouk A.I., Lip G.Y.H., Lavie C.J., Toth P.P., Jukema J.W., Sattar N. (2021). Lipid and Blood Pressure Meta-analysis Collaboration (LBPMC) Group and the International Lipid Expert Panel (ILEP). Association of statin use in older people primary prevention group with risk of cardiovascular events and mortality: A systematic review and meta-analysis of observational studies. BMC Med..

[96] Brach J.S., Solomon C., Naydeck B.L., Sutton-Tyrrell K., Enright P.L., Swords-Jenny N. (2008). Incident physical disability in people with lower extremity peripheral arterial disease: The role of cardiovascular disease. J. Am. Geriatr. Soc..

[97] Paneni F., Beckman J., Creager M.A., Cosentino F. (2013). Diabetes and vascular disease: Pathophysiology, clinical consequences, and medical therapy: Part I. Eur. Heart J..

[98] Velescu A., Clara A., Penafiel J., Grau M., Degano I.R., Ramos R., Ramos R., Marrugat J., Elosua R. (2016). Peripheral Arterial Disease Incidence and Associated Risk Factors in a Mediterranean Population-Based Cohort. The REGICOR Study. Eur. J. Vascul. Endovasc. Surg..

[99] Conen D., Everett B.M., Kurth T., Creager M.A., Buring J.E., Ridker P.M., Pradhan R.D. (2011). Smoking, Smoking Cessation and Risk of Symptomatic Peripheral Artery Disease in Women: A Prospective Study. Ann. Intern. Med..

[100] Olin J.W., White C.J., Armstrong E.J., Kadian-Dodov D., Hiatt W.R. (2016). Peripheral Artery Disease: Evolving Role of Exercise, Medical Therapy, and Endovascular Options. J. Am. Coll. Cardiol..

[101] Hussain M.A., Al-Omran M., Creager M.A., Anand S.S., Verma S., Bhatt D.L. (2018). Antithrombotic Therapy for Peripheral Artery Disease: Recent Advances. J. Am. Coll. Cardiol..

[102] Alexa I.D., Prada G.I., Donca V.I., Mos L.M., Alexa O. Improving Quality of Life of Elderly People Aged 85 and Older by Improving Treatment Adherence. Proceedings of the 4th IEEE International Conference on E-Health and Bioengineering.

[103] Langhammer B., Bergland A., Rydwik E. (2018). The Importance of Physical Activity Exercise among Older People. Bio. Med. Res. Int..

[104] Nied R.J., Franklin B. (2002). Promoting and Prescribing Exercise for the Elderly. Am. Fam. Physician.

[105] Ko G., Davidson L.E., Brennan A.M., Lam M., Ross R. (2016). Abdominal Adiposity, Not Cardiorespiratory Fitness, Mediates the Exercise-Induced Change in Insulin Sensitivity in Older Adults. PLoS ONE.

[106] Stewart J., Manmathan G., Wilkinson P. (2017). Primary prevention of cardiovascular disease: A review of contemporary guidance and literature. JRSM Cardiovasc. Dis..

[107] Tian D., Meng J. (2019). Exercise for Prevention and Relief of Cardiovascular Disease: Prognoses, Mechanisms, and Approaches. Oxid. Med. Cell. Longev..

[108] Mandolesi L., Polverino A., Montuori S., Foti F., Ferraioli G., Sorrentino P., Sorrentino G. (2018). Effects of Physical Exercise on Cognitive Functioning and Wellbeing: Biological and Psychological Benefits. Front. Psychol..

[109] Cheng S.J., Yu H.K., Chen Y.C., Chen C.Y., Lien W.C., Yang P.Y., Hu G.C. (2013). Physical Activity and Risk of Cardiovascular Disease Among Older Adults. Int. J. Gerontol..

[110] NHS Physical Activity Guidelines for Older Adults. https://www.nhs.uk/live-well/exercise/physical-activity-guidelines-older-adults.

[111] Centers for Disease Control and Prevention How Much Physical Activity Do Older Adults Need?. https://www.cdc.gov/physicalactivity/basics/older_adults/index.htm.

[112] Shaked O., Cohen G., Goshen A., Shimony T., Shohat T., Gerber Y. (2021). Physical Activity and Long-Term Mortality Risk in Older Adults with and without Cardiovascular Disease: A Nationwide Cohort Study. Gerontology.

[113] Lachman S., Boekholdt S.M., Luben R.N., Sharp S.J., Brage S., Khaw K.T., Peters R.J., Wareham N.J. (2018). Impact of physical activity on the risk of cardiovascular disease in middle-aged and older adults: EPIC Norfolk prospective population study. Eur. J. Prev. Cardiol..

[114] Izquierdo M., Duque G., Morley J.E. (2021). Physical activity guidelines for older people: Knowledge gaps and future directions. Lancet Health Longev..

[115] Muntaner-Mas A., Vidal-Conti J., Borràs P.A., Ortega F.B., Palou P. (2017). Effects of a Whatsapp-delivered physical activity intervention to enhance health-related physical fitness components and cardiovascular disease risk factors in older adults. J. Sports Med. Phys. Fit..

[116] McGarrigle L., Todd C. (2020). Promotion of Physical Activity in Older People Using mHealth and eHealth Technologies: Rapid Review of Reviews. J. Med. Internet Res..

[117] Filar-Mierzwa K., Długosz-Boś M., Marchewka A., Aleksander-Szymanowicz P. (2021). Effect of different forms of physical activity on balance in older women. J. Women Aging.

[118] Hall K.E., McDonald M.W., Grise K.N., Campos O.A., Noble E.G., Melling C.W.J. (2013). The role of resistance and aerobic exercise training on insulin sensitivity measures in STZ-induced Type 1 diabetic rodents. Metabolism.

[119] Zheng F., Cai Y. (2019). Concurrent exercise improves insulin resistance and nonalcoholic fatty liver disease by upregulating PPAR-γ and genes involved in the beta-oxidation of fatty acids in ApoE-KO mice fed a high-fat diet. Lipids Health Dis..

[120] Kim C.H., Youg J.H., Park J.Y., Hong S.K., Park K.S., Park S.W. (2000). Effects of high-fat diet and exercise training on intracellular glucose metabolism in rats. Am. J. Physiol. Endocrinol. Metab..

[121] Diaz K.M., Schimbo D. (2013). Physical activity and the prevention of hypertension. Curr. Hypertens. Rep..

[122] Carpio-Rivera E., Moncada-Jimenez J., Salazar-Rojas W., Solera-Herrera A. (2016). Acute Effects of Exercise on Blood Pressure: A Meta-Analytic Investigation. Arquivos Brasileiros de Cardiologia..

[123] Tsukiyama Y., Ito T., Nagaoka K., Eguchi E., Ogino K. (2017). Effects of exercise training on nitric oxide, blood pressure and antioxidant enzymes. J. Clin. Biochem. Nutr..

[124] Roque F.R., Briones A.M., Garcia-Redondo A.B., Galan M., Martinez-Revelles S., Avendano M.S., Cachofeiro V., Fernandes T., Vassallo D.V., Oliveira E.D. (2013). Aerobic exercise reduces oxidative stress and improves vascular changes of small mesenteric and coronary arteries in hypertension. Br. J. Pharmacol..

[125] Brito A.F., Silva A.S., Souza I.L.L., Pereira J.C., Da Silva B.A. (2015). Intensity of swimming exercise influences aortic reactivity in rats. Braz. J. Med. Biol. Res..

